# Transcriptional Control of *Trpm6* by the Nuclear Receptor FXR

**DOI:** 10.3390/ijms23041980

**Published:** 2022-02-10

**Authors:** Eun Young Kim, Jae Man Lee

**Affiliations:** 1 Department of Biochemistry and Cell Biology, Cell and Matrix Research Institute, School of Medicine, Kyungpook National University, Daegu 41944, Korea; key11@knu.ac.kr; 2 BK21 FOUR KNU Biomedical Convergence Program, Department of Biomedical Science, Kyungpook National University, Daegu 41944, Korea

**Keywords:** nuclear receptor, FXR, bile acid, *Trpm6*, small intestine, colon, magnesium

## Abstract

Farnesoid x receptor (FXR) is a nuclear bile acid receptor that belongs to the nuclear receptor superfamily. It plays an essential role in bile acid biosynthesis, lipid and glucose metabolism, liver regeneration, and vertical sleeve gastrectomy. A loss of the *FXR* gene or dysregulations of FXR-mediated gene expression are associated with the development of progressive familial intrahepatic cholestasis, tumorigenesis, inflammation, and diabetes mellitus. Magnesium ion (Mg^2+^) is essential for mammalian physiology. Over 600 enzymes are dependent on Mg^2+^ for their activity. Here, we show that the *Trpm6* gene encoding a Mg^2+^ channel is a direct FXR target gene in the intestinal epithelial cells of mice. FXR expressed in the intestinal epithelial cells is absolutely required for sustaining a basal expression of intestinal *Trpm6* that can be robustly induced by the treatment of GW4064, a synthetic FXR agonist. Analysis of FXR ChIP-seq data revealed that intron regions of *Trpm6* contain two prominent FXR binding peaks. Among them, the proximal peak from the transcription start site contains a functional inverted repeat 1 (IR1) response element that directly binds to the FXR-RXRα heterodimer. Based on these results, we proposed that an intestinal FXR-TRPM6 axis may link a bile acid signaling to Mg^2+^ homeostasis.

## 1. Introduction

FXR (also known as NR1H4 and RIP14) is a member of the nuclear receptor superfamily and is primarily expressed in liver, intestine, and kidney. As a heterodimer with retinoid x receptor (RXR), FXR binds to response elements in the regulatory region of target genes to control their transcription. The FXR-RXR heterodimer exhibits a high affinity to bind to an inverted repeat 1 response element (5′-AGGTCA N TGACCT-3′; N is any single nucleotide). Because of the identifications of some bile acids as endogenous agonist or antagonist ligands, FXR has been considered as an adopted orphan nuclear receptor [1,2,3,4,5,6,7,8].

In accordance with its endogenous ligands, FXR plays a pivotal role in bile acid homeostasis and its associated lipid and glucose metabolism [9,10,11,12,13,14,15]. Moreover, it has been shown that FXR is required for normal liver regeneration and beneficial effects of type 2 diabetes and vertical sleeve gastrectomy in mice [16,17,18,19,20]. Loss of FXR is also associated with tumorigenesis of hepatocellular carcinoma and colorectal cancer, progressive familial intrahepatic cholestasis (PFIC), and inflammation [21,22,23,24,25]. As a bile acid sensor in the fed state, FXR in the intestine and liver is suspected to be activated by enterohepatic circulation along with fat-soluble nutrients and vitamins. This concept allowed us to demonstrate that postprandial activation of FXR is necessary for suppressing autophagy, an intracellular degradation process involved in lysosomes [26,27]. Recently, it has also been reported that FXR-mediated *Rubicon* induction impedes autophagy in human cholestatic conditions [28]. Therefore, several academies and pharmaceutical companies have intensively investigated to develop potent synthetic ligands for FXR, aiming to treat metabolic disorders and cancers [29,30,31,32,33]. As a result of these efforts, several synthetic ligands have been developed, which is very useful for unveiling novel FXR target genes. Among them, obeticholic acid (OCA), a semisynthetic chenodeoxycholic aid (also known as 6α-ethyl-chenodeoxycholic acid or INT-747), is about 100-fold more potent than chenodeoxycholic acid (CDCA) and has been approved to treat primary biliary cholangitis despite pruritis as a side effect for some patients [34,35]. Inspired by this, other clinical applications of OCA are currently underway [36].

Magnesium (Mg^2+^) is the second most abundant intracellular cation and is essential for human physiology. Mg^2+^ plays a critical role in various tissues to sustain healthy life. More than 600 enzymatic reactions, including those involved in energy metabolism and protein synthesis, require Mg^2+^ for their activity. Chronic Mg^2+^ deficiency seems to be intimately associated with the development of metabolic diseases such as obesity, hypertension, chronic kidney diseases, cardiovascular diseases, and diabetes mellitus [37,38,39,40]. In contrast, Mg^2+^ supplementation has beneficial effects on the improvement of preeclampsia, migraine, depression, coronary artery disease, and asthma. However, excess intake of Mg^2+^ gives rise to diarrhea, resulting in further Mg^2+^ loss. For a normal adult, the daily intake of Mg^2+^ is about 300–400 mg and about 30–50% is absorbed in the small intestine and colon via transcellular and paracellular pathways [37]. Several forms of hereditary hypomagnesemia in humans have led to the identification of causative genes, including the transient receptor potential melastatin 6 (TRPM6) and cyclin M2 (CNNM2) [41,42,43,44,45].

TRPM6 is a Mg^2+^ channel that has been known to be expressed at the apical membrane of the colon and the distal convoluted tubules (DCT) of the kidney [46]. In contrast, TRPM7, a closest homologue of TRPM6, is also a Mg^2+^ channel that is ubiquitously expressed throughout tissues [47,48]. TRPM6 consists of six transmembrane domains with a por region between the fifth and the sixth segment and a large kinase domain at the C-terminal. Although there is some controversy, TRPM6 may act as homo- or heterotetramers with TRPM7 [49,50].

In addition to dietary Mg^2+^, pH, and ATP, TRPM6 has been shown to be regulated by numerous factors at the levels of gene expression, channel activity, and membrane targeting. For example, estrogens increase its mRNA levels in the kidney [47]. Similarly, a cyanidin-dependent nuclear localization of the nuclear receptor PPARα contributes to the induction of *Trpm6* in the mouse colonic epithelial MCE301 cells [51]. Insulin increases channel activity via CDK5-mediated phosphorylation [52]. Epidermal growth factor promotes TRPM6 to be inserted into the plasma membrane via a PI3K-AKT-RAC1-dependent manner [53].

In this study, we find that *Trpm6* is a direct FXR target gene in mouse intestinal epithelial cells. FXR is necessary for sustaining basal expression of intestinal *Trpm6* in mice. Moreover, pharmacological activation of FXR robustly induces the expression of the *Trpm6* gene in the ileum but not in the colon of mice. By analyzing FXR ChIP-seq results previously performed in mouse intestines [54], we discovered that there are a couple of prominent FXR binding peaks in the intron regions of the *Trpm6* gene. Among them, we demonstrated that a proximal FXR binding peak from a transcription start site was functional for FXR transactivation in cell-based reporter assays, and that this peak contains an IR1 response element that directly binds to a FXR-RXR heterodimer in electrophoretic mobility shift assays. These results reveal an unexpected role of FXR in the regulation of intestinal TRPM6-mediated Mg^2+^ absorption from diets. We proposed that the FXR-TRPM6 axis might link a bile acid signaling to Mg^2+^ homeostasis.

## 2. Results

### 2.1. Nutrient Availability Regulates the Expression of Genes encoding Mg^2+^ Channels, Exchangers, and Transporters

To define whether nutrient availability affects Mg^2+^ homeostasis, we decided to determine the expression levels of genes encoding Mg^2+^ channels, exchangers and transporters. To do this, we harvested several metabolic tissues, including the liver, small intestine, colon, and kidney cortex of wild-type C57BL/6J mice in a normal-chow diet fed, fasted, or refed condition.

**Figure 1:**
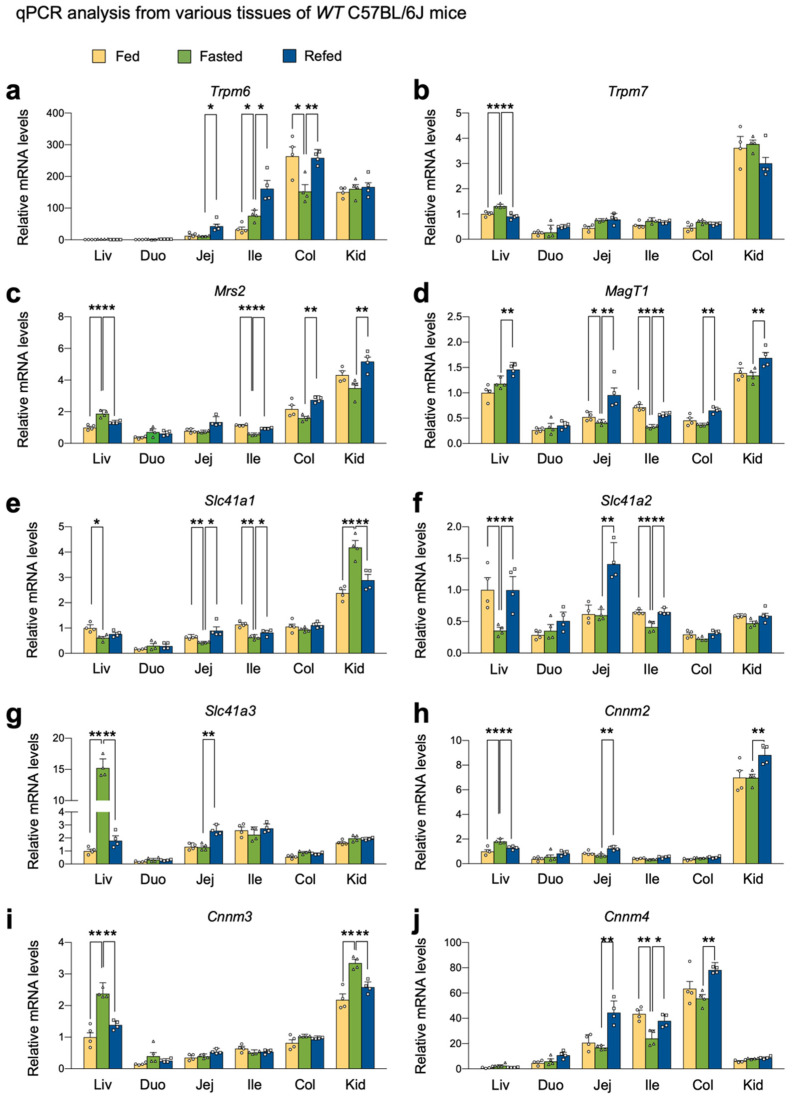
Nutrient availability regulated the expression of genes encoding Mg^2+^ channels, exchangers, and transporters. 8-week-old male wild-type C57BL/6J mice fed ad libitum on a normal-chow diet, fasted for 24 h, or refed for 24 h after 24 h fasting were sacrificed at 17:00 to collect tissues and serum. Expression levels of genes encoding Mg^2+^ channels TRPM6 (**a**), TRPM7 (**b**), MRS2 (**c**), and *MagT1* (**d**), exchangers SLC41A1 to 3 (**e**–**g**), and transporters CNNM2 to 4 (**h**–**j**) were determined in indicated tissues by qPCR analysis. *n* = 4 per group. * *p* < 0.05, ** *p* < 0.01 as analyzed by two-tailed Student’s *t*-test. Data points show individual mice. Data are mean ± s.e.m. WT, wild-type; Liv, liver; Duo, duodenum; Jej, jejunum; Ile, ileum; Col, colon; Kid, kidney cortex.

Consistent with the prior study [55], the colons showed the highest levels of *Trpm6* gene expression in most conditions. However, fasting markedly downregulated its expression; however, this was completely reversed in a refed condition (Figure 1a), indicating that nutrient availability dynamically controls the expression of *Trpm6* gene in the colon. Fasting did not alter *Trpm6* gene expression in the kidney cortex, although its expression was much higher than those in the liver and small intestine (Figure 1a). In contrast to previous reports [47,55], *Trpm6* expression was readily detected in the ileum, where fasting significantly induced its expression. Intriguingly, the refed condition even further elevated *Trpm6* mRNA levels in both jejunum and ileum (Figure 1a). On the other hand, similar to prior studies, *Trpm7* expression was quite ubiquitous despite its highest expression in the kidney cortex. Typically, nutrient perturbations did not affect *Trpm7* mRNA levels in most examined tissues, except in the liver (Figure 1b). These data indicated that *Trpm6* expression was progressively elevated from the duodenum to the colon, and that *Trpm7* expression was relatively unaltered in these tissues. Moreover, refeeding might facilitate intestinal Mg^2+^ absorption via the upregulation of Trpm6 gene.

Mitochondrial RNA splicing 2 (MRS2) is regarded as a primary Mg^2+^ channel located in the membrane of mitochondria that store intracellular Mg^2+^ [56]. Due to its significance for ATP binding, mitochondrial Mg^2+^ concentrations might be important for the activity of the TCA cycle and oxidative phosphorylation [57]. The kidney cortex showed the highest *Mrs2* expression, whereas the small intestine showed relatively low expressions. In the ileum, colon, and kidney cortex, refeeding significantly increased its expression; however, opposite expression patterns were observed in the liver (Figure 1c). The Mg^2+^ transporter 1 (*MagT1*) showed ubiquitous expression patterns of the Mg^2+^ channel in the plasma membrane [37]. It is known that *MagT1* mutations are associated with X-linked human immunodeficiency, and that this plays an important role in cytotoxic functions of NK and CD8^+^ T cells [58,59]. Fasting significantly decreased *MagT1* expression in the jejunum and ileum, which was reversed by refeeding (Figure 1d).

Solute carrier family 41 membrane 1 (SLC41A1) is a Na^+^/Mg^2+^ exchanger located in the plasma membrane, facilitating a Na^+^-dependent Mg^2+^ efflux [60,61]. SLC41A2 and SLC41A3 are the two closest members of SLC41A1, although their functions are less defined [37]. Because of its opposite topology to SLC41A1, SLC41A2 initially proposed to exist in the plasma membrane is suspected to be expressed in organelles and to play an important role in subcellular Mg^2+^ transport [37,62]. We found that fasting notably induced *Slc41a1* expression in the kidney cortex but significantly downregulated it in the liver, jejunum, and ileum (Figure 1e). On the other hand, refeeding markedly increased *Slc41a2* expression in the liver, jejunum, and ileum compared to fasting (Figure 1f). *Slc41a3* is the most highly inducible gene in the fasted liver (Figure 1g).

Members of the cyclin M (CNNM) family have been proposed to be a plasma membrane Mg^2+^ transporter responsible for Mg^2+^ influx [37]. Consistent with prior studies [63], *Cnnm2* showed the highest expression patterns in the kidney (Figure 1h) and *Cnnm3* showed ubiquitous expression patterns throughout tissues (Figure 1i). *Cnnm4* was typically expressed in the intestine (Figure 1j). Fasting significantly increased hepatic and renal *Cnnm3* expressions (Figure 1i) and downregulated *Cnnm4* expression in the intestine (Figure 1j). Refeeding notably increased *Cnnm4* expressions in the intestine (Figure 1j). These results indicated that nutrient availability actively regulated *Cnnm4* expression in the intestine.

### 2.2. FXR Agonism Induces Ileal Trpm6 Expression in Cyp27^-/-^ Mice

**Figure 2:**
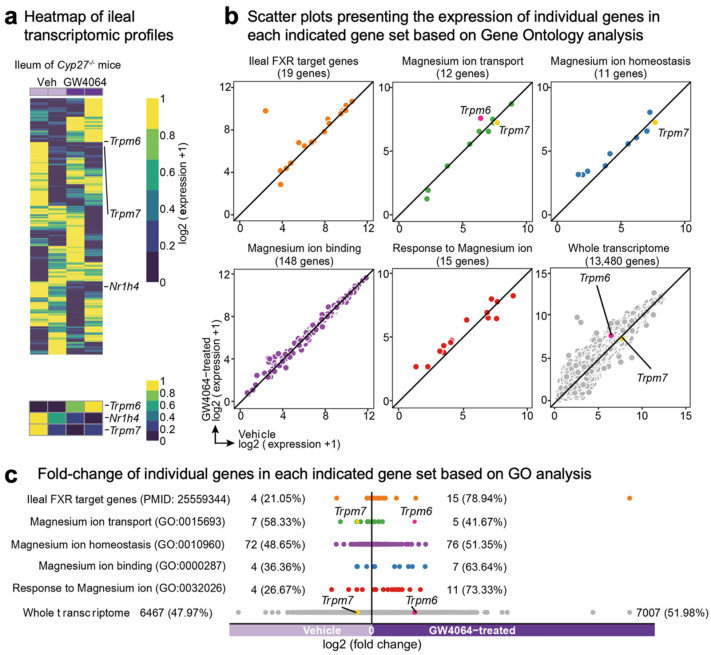
FXR agonism induces *Trpm6* expression in the ileum of *Cyp27^-/-^* mice. (**a**) Heatmap showing the whole ileal transcriptome profile and the expression of *Trpm6*, *Nr1h4* (also known as *Fxrα*), and *Trpm7* in either vehicle or GW4064-administrated *Cyp27^-/-^* mice (NCBI’s Gene Expression Omnibus, accession number GSE40821) [64]. (**b**,**c**) Scatter plots representing the expression (**b**) and fold-change (**c**) of individual genes included in each indicated gene set (Gene Ontology).

Among these genes, previous microarray analysis has shown that treatment of GW4064, a potent synthetic FXR agonist, leads to an about 3-fold induction of the ileal *Trpm6* gene in mice lacking the gene sterol-27-hydroxylase (CYP27), a key enzyme for the alternative (also known as acidic) pathway of bile acid synthesis (NCBI’s Gene Expression Omnibus, accession number GSE40821) [64]. These *Cyp27^-/-^* mice have been reported to produce only low levels of bile acids and, thus, to be substantially devoid of endogenous FXR agonists [65]. Given these features, these mice allowed for discerning GW4064-mediated pharmacological FXR activation from bile acids-mediated physiological FXR activation. Therefore, we decided to reanalyze these microarray data to examine whether GW4064 treatment systematically regulated the transcription of genes involved in Mg^2+^ homeostasis in the ileum of *Cyp27^-/-^* mice. Consistent with a prior study, our heatmap analysis showed that ileal *Trpm6* expression was increased in GW4064-treated mice compared with those of vehicle-treated mice. In contrast, downregulated expression patterns of *Fxr* (also known as Nr1h4) and *Trpm7* were observed in GW4064-treated mice (Figure 2a). Scatter plot analysis also showed that GW4064 treatment upregulated a significant number of genes involved in Mg^2+^ transport, Mg^2+^ homeostasis, Mg^2+^ binding, and response to Mg^2+^ (Figure 2b). Particularly, gene ontology (GO) analysis showed that genes responsive to Mg^2+^ ions were upregulated more than 70% in response to GW4064 (Figure 2c). These results indicated that pharmacological activation of FXR regulated Mg^2+^ homeostasis at the level of transcription.

### 2.3. FXR Is Necessary for Increasing Intestinal Expression of Trpm6 in Response to GW4064

**Figure 3:**
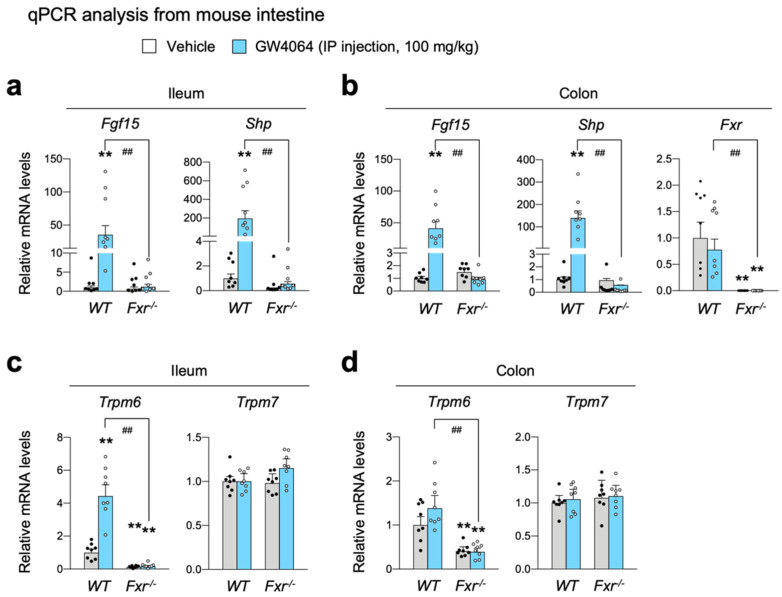
FXR is required for increasing ileal *Trpm6* expression by the treatment of GW4064. Ad libitum fed wild-type and *Fxr^-/-^* mice on a normal chow diet were intraperitoneally injected with vehicle or GW4064 (100 mg/kg BW) twice a day (00:00 a.m. and 12:00 p.m.) for 24 h. *n* = 8 per group. All mice were sacrificed, and tissues were collected at 17:00–18:00. (**a**–**d**) Expression levels of genes encoding FGF15, SHP, FXR, TRPM6, or TRPM7 were determined in the ileum (**a**,**c**) and colon (**b**,**d**) by qPCR analysis. ** *p* < 0.01 vs. wild-type mice treated with vehicle, ^##^ *p* < 0.01 as analyzed by two-tailed Student’s *t*-test. Data points show individual mice. Data are mean ± s.e.m. WT, wild-type; GW, GW4064.

To confirm the bioinformatic analysis shown Figure 2a, wild-type C57BL/6J and *Fxr^-/-^* mice were intraperitoneally treated with GW4064 (100 mg/kg body weight) twice a day. Five hours after the last treatment, the small intestine and colon were harvested to determine FXR activation by qPCR analysis. As expected, in both ileum and colon, GW4064 robustly induced known FXR target genes *Fgf15* and *Shp* (also known as *Nr0b2*) in wild-type mice; however, these inductions were almost completely lost in *Fxr^-/-^* mice (Figure 3a,b), indicating that pharmacological activation of FXR in the intestine was achieved by GW4064 treatment. By observing an almost complete loss of FXR mRNA levels, we also confirmed an ablation of the *Fxr* gene in the intestine of *Fxr^-/-^* mice (Figure 3b).

It is of interest to note that *Trpm6* expression was markedly reduced in both the ileum and colon of *Fxr^-/-^* mice (Figure 3c,d), indicating that FXR is necessary for sustaining a basal *Trpm6* expression in the intestine. As shown in *Cyp27^-/-^* mice, GW4064 markedly increased *Trpm6* expression in the ileum of WT mice, which was completely lost in *Fxr^-/-^* mice (Figure 3c), suggesting that increased expression of ileal *Trpm6* gene upon GW4064 treatment is absolutely dependent on FXR. In contrast to a dynamic regulation of *Trpm6* transcription, *Trpm7* expressions were nearly unaltered by either the loss of FXR or GW4064 treatment (Figure 3b). Consistent with our results, Bijsmans et al. has also reported a similar finding showing that OCA treatment increased *Trpm6* expression in both ileum and intestinal organoids of WT mice but not in those of *Fxr^-/-^* mice [66].

### 2.4. Cell-Autonomous Activation of FXR Is Required for GW4064-Mediated Induction of Trpm6 in Ileal Epithelial Cells

**Figure 4:**
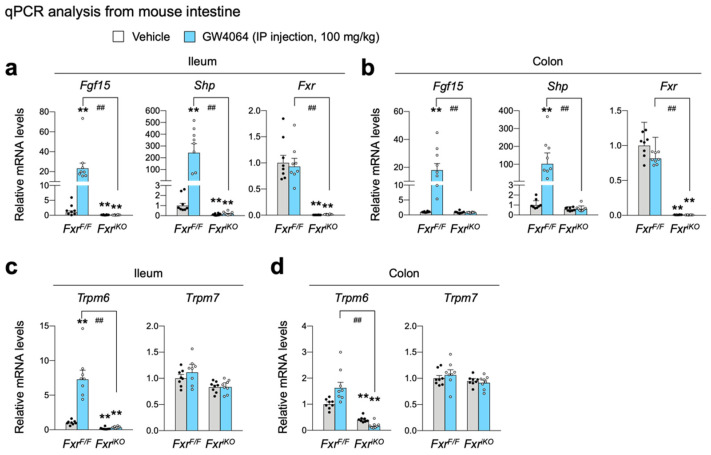
Cell-autonomous activation of FXR in ileal epithelial cells is required for increasing *Trpm6* expression in response to the treatment of GW4064. Ad libitum fed *Fxr^F/F^* mice and *Fxr^iKO^* mice on a normal chow diet were intraperitoneally injected with vehicle or GW4064 (100 mg/kg BW) twice a day (00:00 a.m. and 12:00 p.m.) for a 24 h. *n* = 8 per group. All mice were sacrificed, and tissues were collected at 17:00–18:00. (**a**,**b**) Expression levels of genes encoding FGF15, SHP, FXR, TRPM6, or TRPM7 were determined in the ileum (**a**,**c**) and colon (**b**,**d**) by qPCR analysis. ** *p* < 0.01 vs. wild-type mice treated with vehicle, ^##^ *p* < 0.01 as analyzed by two-tailed Student’s *t*-test. Data points show individual mice. Data are mean ± s.e.m. WT, wild-type; GW, GW4064.

To define whether FXR in intestinal epithelial cells is also required for the regulation of *Trpm6* expression, we generated intestinal epithelial cell-specific Fxr knockout (*Fxr^iKO^*) mice by crossing male Villin1-Cre mice with female *Fxr^F/F^* mice. Similar to Figure 3, control littermate *Fxr^F/F^* and *Fxr^iKO^* mice were intraperitoneally treated with GW4064 twice a day, followed by harvesting small intestine and colon to prepare total RNA (Figure 4). Specific *Fxr* ablations in the intestinal epithelial cells were confirmed by qPCR analysis, showing a complete loss of *Fxr* mRNA levels in *Fxr^iKO^* mice (Figure 4a,b). Expression levels of ileal FXR target genes *Fgf15* and *Shp* were also significantly decreased in *Fxr^iKO^* mice (Figure 4a). Similar to *Fxr^-/-^* mice shown in Figure 3a,b, GW4064 treatment markedly increased the expressions of intestinal FXR target genes *Fgf15* and *Shp* in control *Fxr^F/F^* mice but these responses were completely absent in *Fxr^iKO^* mice (Figure 4a,b).

As with *Fxr^-/-^* mice (Figure 3c,d), *Trpm6* expression in the ileum and colon was significantly downregulated in Fxr^iKO^ mice (Figure 4c,d), indicating a cell-autonomous requirement of FXR in intestinal epithelial cells to maintain a basal *Trpm6* expression. Similar to the results shown in Figure 3c, GW4064 treatment in control *Fxr^F/F^* mice significantly elevated ileal *Trpm6* expression but not colonic *Trpm6* expression. Consistently, GW4064-mediated induction of these ileal *Trpm6* expressions was completely lost in *Fxr^iKO^* mice. Similar to results shown in Figure 3d, both ileal and colonic *Trpm7* expressions were not changed by the loss of FXR in the intestinal epithelial cells, and the treatment of GW4064 (Figure 4d). Taken together, these results suggest that FXR in the intestinal epithelial cells is necessary for the basal *Trpm6* expression in the intestine, and that pharmacological activation of FXR is sufficient for induction of ileal *Trpm6* expression. Based on these findings, we concluded that GW4064-mediated induction of ileal *Trpm6* expression is dependent on intestinal epithelial FXR.

### 2.5. Identification of Trpm6 as a Direct FXR Target Gene in Mouse Intestine

**Figure 5:**
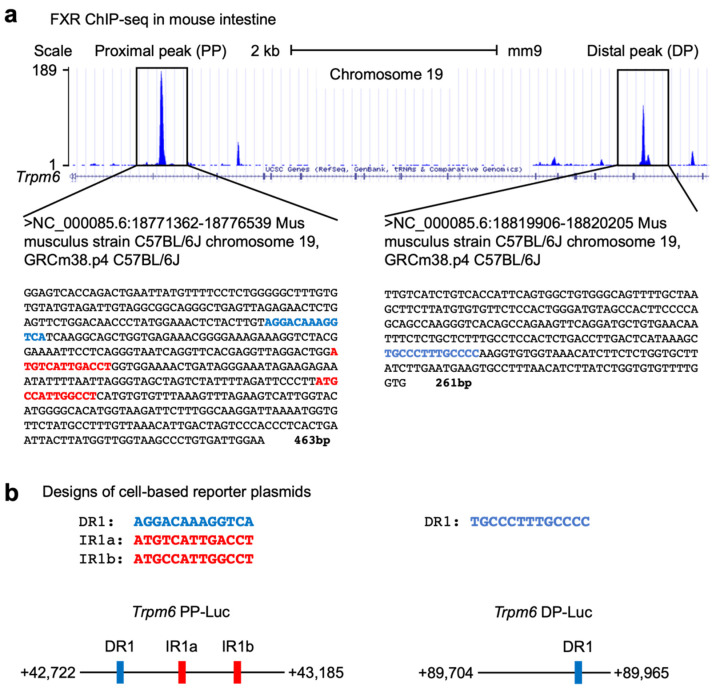
Identification of *Trpm6* as a direct FXR target gene in mouse intestine. (**a**) Mouse genome browser track of the *Trpm6* locus from intestinal FXR ChIP-seq performed previously by the Guo laboratory [54]. A proximal peak (PP, left-side box) and a distal peak (DP, right-side box) represent FXR binding sites in mouse intestine. A bioinformatic analysis using the NHR-scan [67] found that the PP encompassing 463 bp contains one direct repeat 1 (denoted by blue-colored letters) and two inverted repeat 1 response elements (IR1a and IR1b, denoted as red-colored letters), and that the DP encompassing 261 bp has one direct repeat 1 response element (denoted by blue-colored letters). (**b**) Schematic diagrams of cell-based reporter constructs for PP (left) or DP (right) regions of mouse Trpm6 gene. Data are mean ± s.e.m. DR1, direct repeat 1; IR1, inverted repeat 1.

To investigate whether Trpm6 is a direct FXR target gene, we analyzed FXR-ChIP seq data sets performed in the mouse intestine by the Guo laboratory [54]. We found that there were a couple of prominent FXR binding peaks in the intron regions of the *Trpm6* gene. Among them, we focused on two major peaks, a proximal peak (PP, +42,722 to +43,185) and a distal peak (DP, +89,704 to +89,965) (Figure 5a). A bioinformatic analysis of these sequences using the NHR-scan program [67] allowed us to identify that the proximal peak contains one DR1 response element and two IR1 response elements (denoted by IR1a and IR1b), and that the distal peak contains one DR1 response element (Figure 5a). It is of interest to note that the distal peak only contains a DR1 response element instead of an IR1 response element. Nevertheless, it has also been reported that FXR binds DR1 response elements in the promoter regions of human *APOC3* and *APOA* genes and in the enhancer regions of mouse autophagy-related genes, where it functions as a negative transcriptional repressor [26,68,69].

To determine whether these peaks of the mouse *Trpm6* gene are functionally important for FXR transactivation, we have initially generated two luciferase reporter constructs, *Trpm6* PP-Luc containing DNA sequences of a proximal peak and *Trpm6* DP-Luc containing DNA sequences of a distal peak (Figure 5b).

### 2.6. Identification of an IR-1 Response Element for FXR Transactivation in Mouse Trpm6 Gene

**Figure 6:**
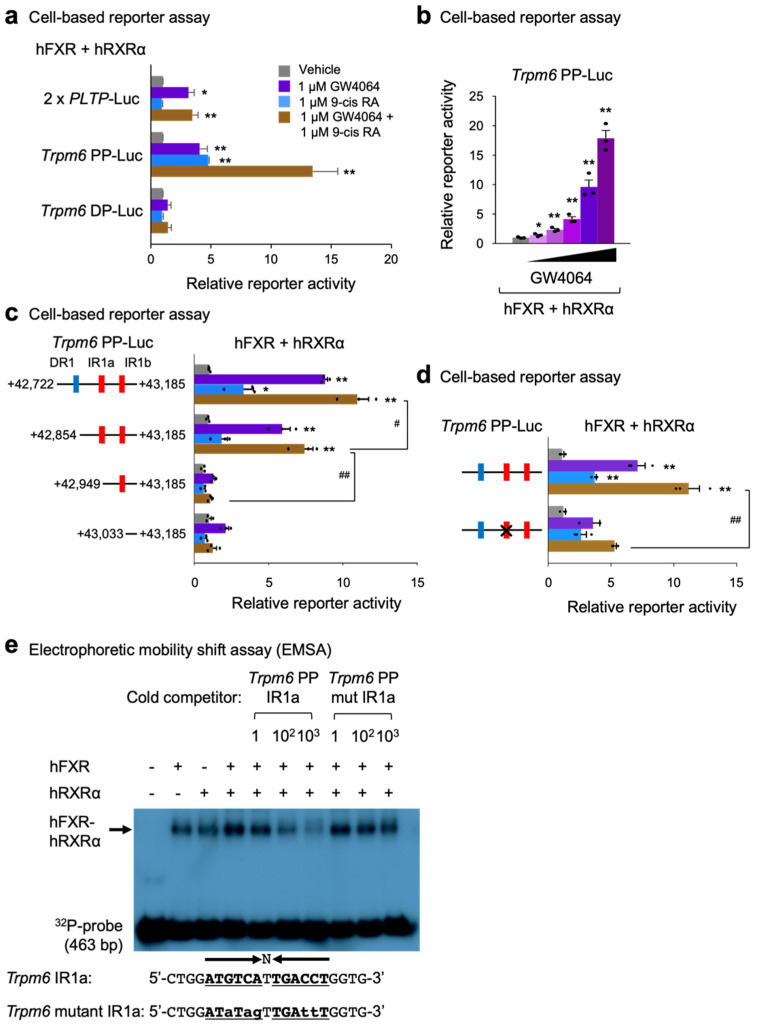
Identification of an IR1 response element for FXR transactivation in mouse *Trpm6* gene. (**a**) Cell-based reporter assays in HeLa cells co-transfected with the indicated luciferase reporter constructs (2 X *PLTP*-Luc, *Trpm6* PP-Luc, or *Trpm6* DP-Luc) and pCMX-β-galactosidase in the presence of FXR and RXRα expression plasmids (pCMX-FXR or pCMX-RXRα). Cells were treated with either vehicle (0.1% DMSO, gray bars,), GW4064 (1 μM, purple bars), 9-cis RA (1 μM, blue bars) or both (brown bars) for 24 h. Three independent experiments were performed. (**b**) Cell-based reporter assays in HeLa cells co-transfected with the *Trpm6* PP-luciferase reporter construct (*Trpm6* PP-Luc) as shown in panel (**a**). Cells were treated with either vehicle (gray bars, 0.1% DMSO) or GW4064 (purple bars) in a dose-dependent manner (GW4064: 1 nM, 10 nM, 100 nM, 1 μM, and 10 μM) for 24 h. Three independent experiments were denoted as black-colored dots. (**c**) Cell-based reporter assays in HeLa cells co-transfected with the depicted luciferase reporter constructs as shown in panel (**a**). Normalized values (luciferase activity/β-galactosidase activity) of vehicle-treated cells were set as fold 1. (**d**) Cell-based reporter assays in HeLa cells co-transfected with the indicated luciferase reporter constructs (control *Trpm6* PP-Luc or mutant *Trpm6* PP-Luc containing point mutations in IR1a site denoted by X) as shown in panel (**a**). (**e**) Electrophoretic mobility shift assays (EMSA) were performed with ^32^P-labeled oligonucleotide sequences containing the *Trpm6* IR1a site extending from +42,722 bp to +43,185 bp in the presence of in vitro transcribed and translated human FXR, human RXRα or both proteins as indicated. Arrow indicates the position of the FXR-RXRα heterodimeric complex. Cold-competitors of the *Trpm6* IR1a sequence with or without mutations, as shown in lowercase alphabet letters, were included at a 1-fold, 100-fold, or 1000-fold molar excess as indicated. * *p* < 0.05, ** *p* < 0.01 vs. each luciferase reporter construct treated with vehicle, ^#^ *p* < 0.05, ^##^ *p* < 0.01 as analyzed by two-tailed Student’s *t*-test (**a**–**d**). Data are mean ± s.e.m. DR1, direct repeat 1; IR1, inverted repeat 1; RA, retinoic acid.

To test functionalities of these constructs, cell-based reporter assays were performed in HeLa cells transiently transfected with reporter constructs and expression plasmids of human FXR and RXRα, as shown in Figure 6a. Sixteen hours after transfection, these cells were then treated with a nonsteroidal synthetic FXR agonist GW4064, an RXR agonist 9-cis retinoic acid, or both for 24 h. We used the 2 × *PLPT*-Luc construct as a positive control reporter plasmid for assessing FXR transactivation [70]. As previously reported, GW4064 treatment significantly increased luciferase activities in 2 × *PLPT*-Luc transfected cells. In *Trpm6* PP-Luc transfected cells, treatments of each agonist ligand significantly elevated luciferase activities and co-treatments of both ligands even further increased luciferase activities. However, these responses were completely absent in *Trpm6* DP-Luc-transfected cells. These data indicate that response elements within the proximal peak of the *Trpm6* gene play a critical role for FXR transactivation. Moreover, we found that luciferase activities of the *Trpm6* PP-Luc construct were gradually increased by GW4064 treatment in a dose-dependent manner (Figure 6b).

To further pinpoint which response elements within the proximal peak region are necessary for FXR transactivation, we generated serial deletion mutant constructs and then performed cell-based reporter assays again (Figure 6c). We found that a deletion construct of DR1 (+42,865 to +43,185) exhibited about 60% of maximal luciferase activity shown in full-length of *Trpm6* PP-Luc construct upon co-treatments of both agonists. However, further deletion of IR1a (+42,949 to +43,185) almost completely abolished these luciferase activities, which were compatible to a reporter construct without these response elements. These data indicate that the IR1a response element within the proximal peak plays an essential role in the FXR transactivation. The importance of this response element was further supported by a mutant Trpm6 PP-Luc construct containing a site-directed mutagenized IR1a site. In cell-based reporter assays, introducing point mutations in the IR1a sequences significantly diminished FXR transactivation upon co-treatments of GW4064 and 9-cis RA, compared with those of the control TRPM6 PP-Luc plasmid (Figure 6d). Taken together, these results clearly demonstrate that the IR1a site of the Trpm6 proximal promoter region is, indeed, functionally important for FXR transactivation.

Finally, to examine whether the IR1a response element directly binds to the FXR-RXRα heterodimer protein, we performed electrophoretic mobility shift assays (EMSA). As expected, the FXR-RXRα heterodimer complex showed the strongest binding to the ^32^P-labeled IR1a probe, although either FXR or RXRα alone could also bind this probe (Figure 6e). Competition analysis showed that an unlabeled cold competitor of IR1a oligonucleotide sequences was able to compete for binding at a 100- or 1000-fold molar excess. Furthermore, the cold mutant competitors of the IR1a oligonucleotide sequences (*Trpm6* mutant IR1a) were almost unable to compete for binding at a 1-, 100-, or 1000-fold molar excess. These results demonstrated that the FXR-RXRα heterodimer could bind specifically to the IR1a located between nucleotides +42,936 and +42,948 in the *Trpm6* gene (Figure 6e). We concluded that this IRa1 response element was required for the FXR and GW4064 activation of the *Trpm6* gene.

## 3. Discussion

FXR is a nuclear bile acid receptor activated in the intestine and liver responding to enterohepatic circulations after postprandial conditions. Because of this, FXR has also been considered as a nutrient-sensing nuclear receptor that is required for suppressing autophagy in a fed state [26,27,71,72,73]. Therefore, first, we wanted to investigate whether nutrient availability can regulate the expression of genes encoding Mg^2+^ transporters, exchangers, or transporters in several metabolic tissues including the liver, duodenum, jejunum, ileum, colon, and kidney cortex. Our results showed that many genes associated with Mg^2+^ homeostasis were altered by either a fasting or refeeding condition. For instance, colonic *Trpm6* and hepatic *Slc41a2* expressions were shown to be repressed by fasting, which was completely reversed by refeeding (Figure 1a,f). Opposite expression patterns were also observed in hepatic *Slc41a3* and *Cnnm3* genes and renal *Slc41a1* gene (Figure 1e,g,i). In contrast to prior studies [47,55], we could detect a significant mRNA level of *Trpm6* in the ileum. Overall, fasting or refeeding could change expression patterns of Mg^2+^-associated genes. Although expressions of many genes were altered in this study, we mainly focused on the regulation of *Trpm6* gene expression by FXR and its synthetic agonist GW4064, due to prior studies [64,66].

Next, we characterized the role of FXR and its synthetic agonist GW4064 in the regulation of intestinal *Trpm6* gene expression. A previous microarray analysis showed that GW4064 treatment resulted in the induction of the ileal *Trpm6* gene in *Cyp27^-/-^* mice [64]. Similarly, it has been reported that OCA treatment increased *Trpm6* expression in the ileum and intestinal organoids of wild-type mice, but that these responses were lost in those of *Fxr^-/-^* mice [66]. These results allowed us to determine whether GW4064 could also regulate *Trpm6* gene expression in vivo. To address this, we treated wild-type mice and *Fxr^-/-^* mice with GW4064 twice a day and observed an at least 4-fold induction in the ileal *Trpm6* mRNA level of wild-type mice. Consistent with previous findings, these inductions were completely lost in *Fxr^-/-^* mice. Moreover, the ileum and colon in *Fxr^-/-^* mice showed a markedly reduced expression of *Trpm6* (Figure 3), suggesting that FXR sustains the basal expression of intestinal *Trpm6* expression. Our further analysis has defined an essential role of FXR expression in the intestinal epithelial cells for the basal *Trpm6* gene expression. We observed a dramatic reduction of *Trpm6* mRNA levels in the ileum and colon of *Fxr^iKO^* mice (Figure 4). It is of interest to note that GW4064 never induced colonic Trpm6 gene expression in wild-type mice and control *Fxr^F/F^* mice (Figure 3d and Figure 4d). These results suggested that GW4064 treatment regulated expression of a given FXR target gene in a tissue-specific manner. Our bioinformatic analyses based on previous FXR ChIP-seq data [54] also showed that the intestinal *Trpm6* gene contained two prominent FXR binding peaks in the intron regions. The proximal peak contained two IR1 response elements (Figure 5) and the FXR-RXRα heterodimer could regulate one of these elements in the cell-based reporter assays (Figure 6a–c). We also demonstrated that this functional IR1 response element could directly bind to the FXR-RXRα heterodimer in EMSA experiments (Figure 6d).

It has been shown that mutations of *TRPM6* in humans is causative for hypomagnesemia with secondary hypocalcemia [42,43]. Subsequently, TRPM6 has been proposed to function as a Mg^2+^ channel for transcellular Mg^2+^ uptake in the colon and Mg^2+^ reabsorption in the DCT segment of the kidney [46]. Physiological roles of TRPM6 have been further examined by generating knockout mice. Most *Trpm6* knockout (*Trpm6^-/-^*) mice showed an embryonic lethality by E12.5, whereas survived mice had severe neural tube defects. Mg^2+^-supplementation to dams marginally improved offspring survival to weaning [74]. Heterozygous *Trm6^+/-^* mice have also been shown to be mild hypomagnesemia with low serum Mg^2+^ levels [75]. Moreover, several conditional knockout mice revealed tissue-specific functions of TRPM6. During embryonic development, TRPM6 plays an essential role in the placenta and yolk sac. In the adult stage, intestinal TRPM6 is necessary for maintaining Mg^2+^ balance but renal TRPM6 is not. Loss of *Trpm6* in adult mice showed reduced lifespan, growth retardation, and metabolic dysfunctions whose phenotypes were rescued by Mg^2+^ supplementation [55]. This literature strongly suggests that colonic TRPM6 is essential for transcellular Mg^2+^ absorption from dietary Mg^2+^.

Prior studies have also reported that the *Trpm6* gene is regulated at the transcriptional levels. Dietary restriction or supplementation of Mg^2+^ has been shown to actively upregulate expressions of *Trpm6* in the kidney or colon, respectively. Moreover, renal *Trpm6* mRNA levels were significantly downregulated in ovariectomized rats, which was rescued by the treatment of 17β-estradiol [47]. These data indicate that the renal *Trpm6* expression might be controlled by the steroid hormone receptors ERα and ERβ. The nuclear receptor PPARα has also been shown to regulate colonic *Trpm6* expressions, at least in mouse colonic epithelial MCE301 cells. Cell-based reporter assays have demonstrated that the promoter region between nucleotides −1214 to −718 of the mouse *Trpm6* gene are functionally important for PPARα transactivation. ChIP-qPCR results have shown that PPARα can be recruited to this region [51]. Taken together, our current findings with the previous literature suggest that several nuclear receptors dynamically regulate *Trpm6* gene expression to control Mg^2+^ homeostasis.

Further investigations might be needed to define whether chronic supplementation or depletion of bile acids affect Mg^2+^ homeostasis, and whether bile acid ligands for FXR agonism or antagonism also dynamically regulate *Trpm6* gene expression in the intestine and kidney.

## 4. Materials and Methods

### 4.1. Chemicals and Reagents

Wild-type C57BL/6J mice were purchased from Japan SLC, Inc. (Hamamatsu, Japan) (C57BL/6JJmsSlc); *Fxr^-/-^* and *Villin1-Cre* mice were purchased from Jackson Laboratory (Sacramento, CA, USA) (strain #004144 and #021504); *Fxr^-/-^* and homozygous Fxr floxed (*Fxr^F/F^*) mice were previously described [9,76]. HeLa cells were purchased from ATCC (CCL-2); GW4064 (Cat.# 2473) from Tocris (Bristol, UK); obeticholic acid (OCA, Cat.# AG-CR1-3560-M025) from Adipogen (San Diego, CA, USA); polyethylene glycol 400 (PEG 400, Cat.# P3265-1KG), Tween 80 (Cat.# P1754-500ML), trizma phosphate (Cat.# T-8655), adenosine triphosphate (ATP, Cat.# A7699), magnesium chloride (Cat.# 208337), and dipotassium phosphate (K_2_HPO_4_, Cat.# P3786), 9-cis retinoic acid (Cat.# R4643) from Sigma-Aldrich (St. Louis, MO, USA); HyClone DMEM high glucose (Cat.# SH30243.01) and fetal bovine serum (FBS, Cat.# SH20084.03) from HyClone (Logan, UT, USA); penicillin-streptomycin (Cat.# 15140122) from Gibco (Carlsbad, CA, USA); Lipofectamine 2000 (Cat.# 11668019) and Trizol Reagent (Cat.# 15596018) from Invitrogen (Waltham, MA, USA); RbTaq^TM^ qPCR 2X PreMIX (SYBR Green with high ROX, Cat.# RT531M) from Enzynomics (Daejeon, Korea); PrimeScript^TM^ 1st strand cDNA Synthesis kit (Cat.# 6110A) and T4 polynucleotide kinase (1000 U, Cat.#2021A) from TaKaRa (Kyoto, Japan); Dimethyl sulfoxide (DMSO, Cat.# sc-358801) from Santa Cruz (Dallas, TX, USA); galacton-plus substrate I 100 X concentrate (Cat.# T218) from Applied Biosystems (Waltham, MA, USA); ACCELERATOR-II 210 ML (Cat.# T2222) from Tropix; D-Luciferin (Cat.# L-8240) from biosynth (Staad, Switzerland); ^32^P-fATP (0.25mCi, Cat.# NEG502A) from Perkin Elmer (Yokohama, Japan); TnT Quick Coupled Transcription/Translation Systems (Cat.# L1170) from Promega (Chuo City, Tokyo); SacIHF (Cat.#R3156S) and BglII (Cat.# R0144S) from NEB (Ipswich, MA, USA); QuickChange Site-Directed Mutagenesis Kit (Cat.# 200519) from Agilent Technologies (Tokyo, Japan). Information for other reagents not shown here is described in the relevant methods and references.

### 4.2. Animal Experiments

All animal studies and procedures were approved by the institutional Animal Care and Use Committee of the Kyungpook National University (KNU-2020-036). *Fxr^F/F^* mice were a gift from Johan Auwerx (Ecole Polytechnique Federale de Lausanne, Switzerland). Male *Villin1-Cre* mice were bred with female *Fxr^F/F^* mice to generate *Villin1-Cre*; *Fxr^F/F^* (*Fxr^iKO^*) mice, which showed an intestinal epithelial cell-specific *Fxr* ablation. All experiments were performed in ad libitum fed male mice unless otherwise indicated. Eight to nine-week-old male wild-type C57BL/6J, *Fxr^-/-^*, *Fxr^F/F^*, and *Fxr^iKO^* mice were intraperitoneally injected with vehicle (0.1% dimethylsulfoxide (DMSO) in 90:5:5 of saline, PEG-400 and Tween 80, respectively) or GW4064 (100 mg/kg body weight) twice a day (first injection at 00:00 and second injection at 12:00). After 5 h of the second injection, mice were sacrificed to collect tissues including livers, intestines (duodenum, jejunum, ileum, and colon), and kidneys. Collected tissues were immediately frozen in liquid nitrogen for molecular studies. To avoid circadian issues, all mice were sacrificed at 17:00–18:00.

### 4.3. Transcriptomic Analysis and Visualization

Transcriptomic analysis and visualization, including a heatmap showing the whole ileal transcriptome profile of either vehicle or GW4064-administrated *Cyp27^-/-^* mice (NCBI’s Gene Expression Omnibus, accession number GSE40821), scatter plot presenting the expression, and fold-change of individual genes included in each indicated gene set (Gene Ontology), were conducted as described previously [77]. Analysis and visualization of the ileal transcriptome of either vehicle or GW4064-administrated *Cyp27^-/-^* mice were obtained from the Gene Expression Omnibus of the National Center for Biotechnology (https://www.ncbi.nlm.nih.gov/geo/; accessed on 11 November 2021) under accession number GSE40821 [64]. All plots were generated with Rstudio (RStudio Desktop 1.4.1717; R 4.1.2), and installed R packages dplyr, stringr, ggpubr, ggplot2, pheatmap, igraph, ggraph, corrr, corrplot, tidyverse, and reshape2 (https://www.r-project.org; accessed on 11 November 2021).

### 4.4. RNA Purification, cDNA Synthesis, and qPCR Analysis

Total RNA was isolated from snap-frozen tissues, including liver, duodenum, jejunum, ileum, colon, and kidney cortex, using Trizol Reagent and prepared for complementary DNA using PrimeScript^TM^ 1st strand cDNA Synthesis kit (Takara). Gene expression was determined by qPCR using RbTaq^TM^ qPCR 2X PreMIX (SYBR Green with high ROX, Enzynomics). mRNA levels were normalized by the *36B4* (also known as *Rplp0*) gene. qPCR primer information is listed in Table S1.

### 4.5. Molecular Cloning

Genomic DNAs encompassing a proximal FXR binding peak (PP, +42,722 to +43,185) or a distal FXR binding peak (DP, +89,704 to +89,965) found in mouse *Trpm6* gene were amplified from the tail genomic DNA of wild-type C57BL/6J mice by the PCR method. Purified DNAs were cloned into pTK-luc plasmid by a serial digestion with SacI and BglII. Serial deletion constructs (+42,854 to +43,185, +42,949 to +43,185, and +43,033 to +43,185) were subsequently prepared in a similar manner as describe above. The IR1a site of the *Trpm6* PP-Luc construct was mutated using the QuickChange Site-Directed Mutagenesis Kit (Agilent Technologies). This mutant was generated using the following oligonucleotide: 5′-TAGGACTGGATaTagTTGAttTGGTGGAAAA-3′. The underline indicates an IR1 response element. Successful cloning was confirmed by DNA sequencing analysis. Oligonucleotide sequences used for constructing luciferase reporter plasmids are listed in Table S2.

### 4.6. Cell-Based Reporter Assays

HeLa cells were maintained in the following media: DMEM high glucose supplemented with 10% FBS, and 1% penicillin/streptomycin antibiotics. For the luciferase assays, HeLa cells were cultured in 24-well plates. Transient transfections were performed using Lipofectamine 2000. Cells were transfected with 200 ng of reporter constructs (2 X *PLTP*-Luc, *Trpm6* PP-Luc, or *Trpm6* DP-Luc), 100 ng of cytomegalovirus-promoter (CMX)-human FXR, CMX-human RXRα, or both, and 50 ng of CMX-β-galactosidase. pCDNA3.1 was added to prepare the total DNA to 500 ng per well. After 16 h transfection, cells were treated with vehicle (0.1% DMSO), 1 μM GW4064, 1 μM 9-cis retinoic acid (RA), or a combination of GW4064 and 9-cis RA. Luciferase and β-galactosidase assays were performed 24 h after drug treatment. Luciferase activity was normalized with β-galactosidase activity. Normalized values from vehicle-treated cells were set as fold 1.

### 4.7. Electrophoretic Mobility Shift Assays

Double-stranded probes corresponding to a *Trpm6* PP region (+42,722 bp to +43,185) were prepared by a PCR method. Double-stranded oligonucleotides of the following sequences were used as unlabeled cold competitors: *Trpm6* IR1a, 5′-CTGGATGTCATTGACCTGGTG-3′ (+42,932 to +42,952), IR1 sequence is denoted by the underlines; *Trpm6* mutant IR1a, 5′-CTGGATaTagTTGAttTGGTG-3′, lowercase letters indicate mutations of IR1 sequence. Double-stranded probe was end-labeled with 1 ng of [χ-^32^P]-ATP (Perkin Elmer, NEG502A) using T4 polynucleotide kinase (TAKARA, 2021A) by incubating them at 37 °C for 30 min followed by heat inactivation at 75 °C for 15 min. Using TnT Quick Coupled Transcription/Translation systems (Promega), in vitro transcribed/translated human FXR and human RXRα were generated from their mammalian expression plasmids. The labeled probe was incubated with 5 μL of in vitro transcribed/translated human FXR, human RXRα, or both proteins at 37 °C for 30 min in a buffer solution (20 mM Tris-HCl, pH 8.0, 15 mM MgCl_2_, 100 mM KCl, 1mM DTT, 100 ng/μL BSA). 6 X DNA dye was added to the mixtures, which were then loaded onto a 6% nondenaturing polyacrylamide gel. One hour after electrophoresis at 100 V in 1 X TBE buffer, gels were dried and subjected to autoradiography by the exposure of X-ray films at −80 °C for overnight. Cold competitors were added in 100- or 1000-fold molar excess to the labeled probe.

### 4.8. Statistical Analysis

All values are shown as mean ± s.e.m. and error bars were derived from biological replicates rather than technical replicates. Significant differences between two groups were evaluated using a two-tailed, unpaired *t*-test, which was found to be appropriate, as groups displayed a normal distribution and comparable variance; *p* < 0.05 was considered statistically significant.

## 5. Conclusions

**Figure 7:**
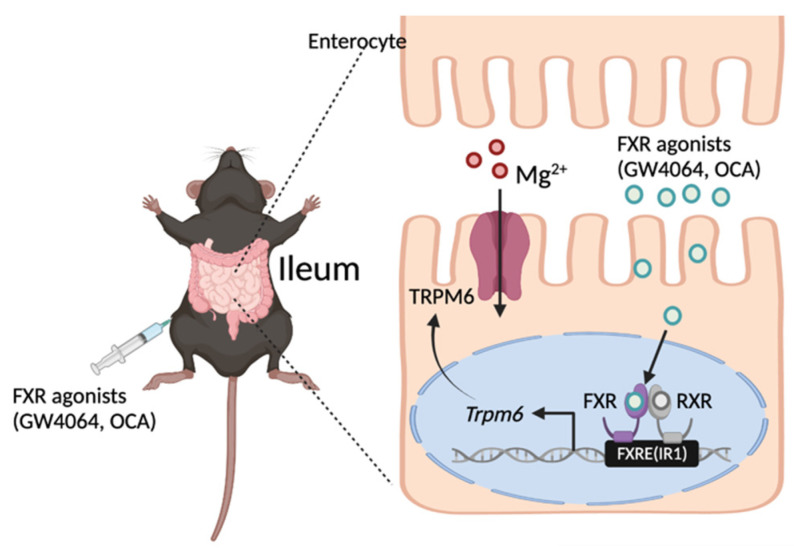
Working model of FXR-mediated *Trpm6* expression in the small intestine. FXR directly binds to an inverted repeat 1 (IR1) response element in the regulatory region of mouse *Trpm6* gene. As a result, FXR in the intestinal epithelial cells is required for maintaining basal expressions of *Trpm6* in mice. Additionally, pharmacological activation of FXR using GW4064 or OCA is sufficient to increase expressions of *Trpm6* in the ileum, but not in the colon of the mice. OCA, obeticholic acid; FXRE, FXR response element. A schematic diagram was created in BioRender.com.

In the present study, we found that FXR in the intestinal epithelial cells was required for maintaining basal expression of *Trpm6* in mice, and that the robust induction of ileal *Trpm6* expression in response to GW4064 was completely dependent on FXR. GW4064 increased reporter activity in the proximal promoter region of *Trpm6* that contains a notable FXR binding peak. Within this peak, FXR directly bound to the IR1a sequence. Therefore, pharmacological activation of FXR might increase TRPM6-mediated Mg^2+^ influx in the ileum, contributing to the energy metabolism of the fed state. This may pave the way for developing therapeutic strategies against metabolic disorders (Figure 7).

## Data Availability

Not applicable.

## Supplementary Materials

The following supporting information can be downloaded at: https://www.mdpi.com/article/10.3390/ijms23041980/s1.
