# Supplementary figures and images for: Transcriptional Control of Trpm6 by the Nuclear Receptor FXR
Source: Int J Mol Sci. 2022 Feb 10;23(4):1980. doi: 10.3390/ijms23041980 (PMC8874704; doi:10.3390/ijms23041980)

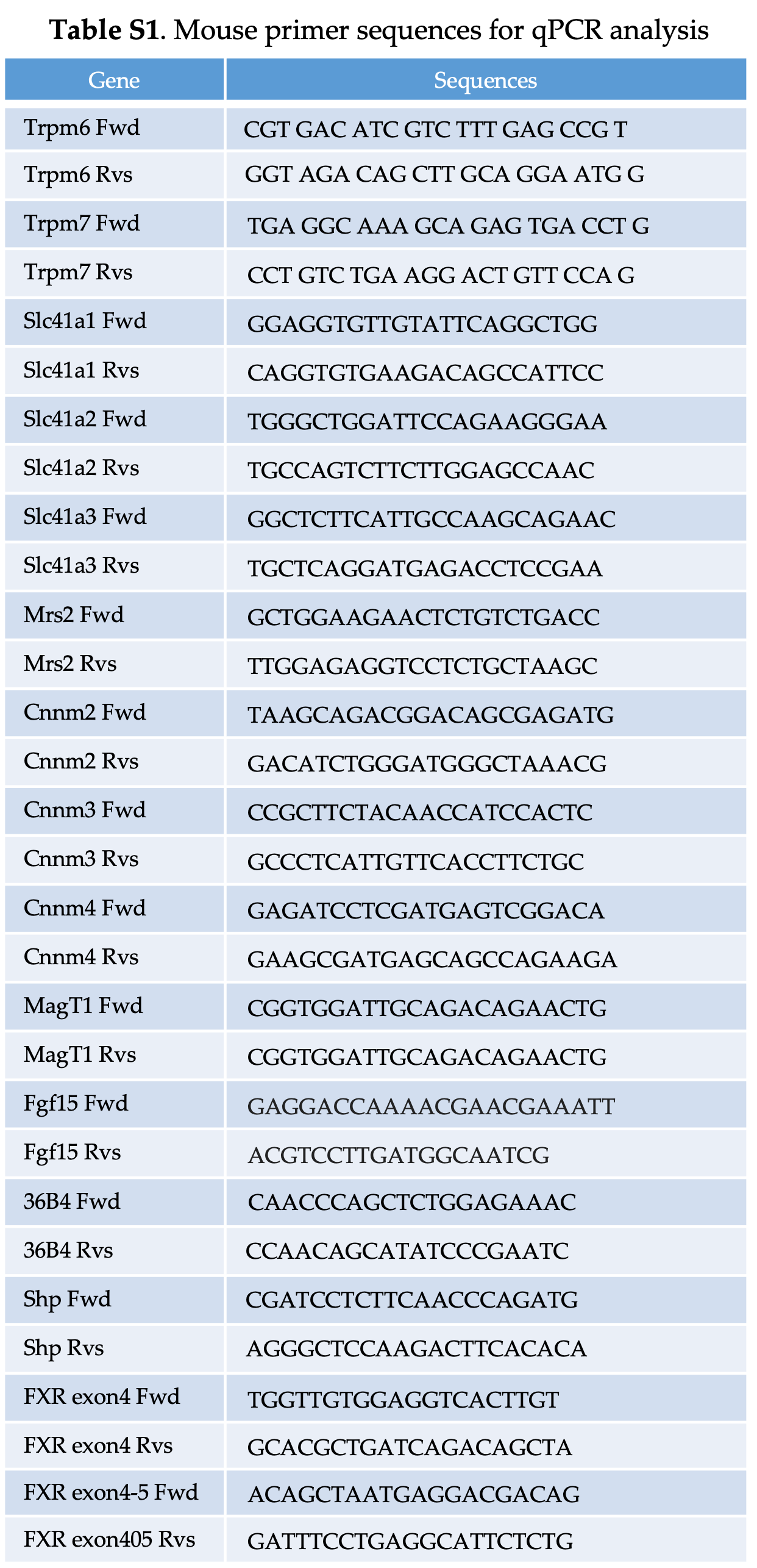

Supplement: Supplementary file 1 [file ijms-23-01980-s001.zip › Table S1.png]

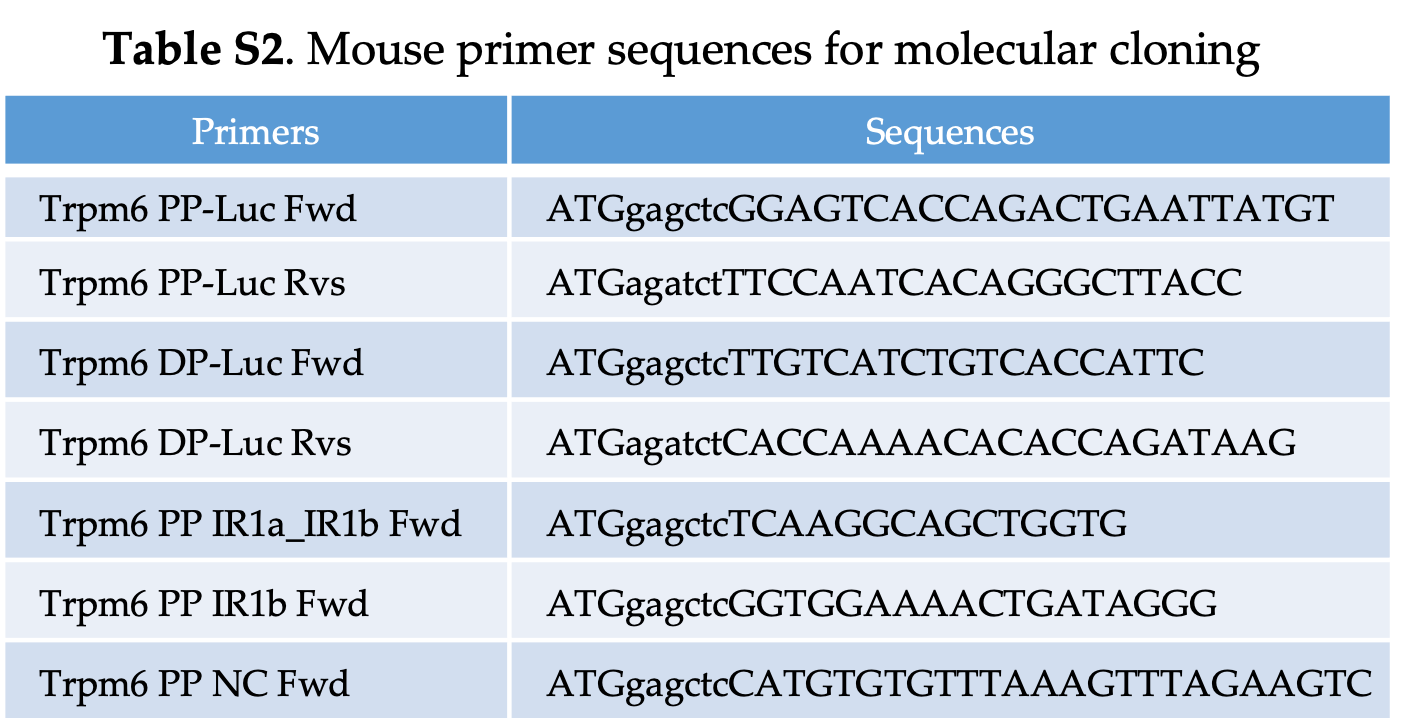

Supplement: Supplementary file 1 [file ijms-23-01980-s001.zip › Table S2.png]
